# Prospectively assessing risk for premature ovarian senescence in young females: a new paradigm

**DOI:** 10.1186/s12958-015-0026-z

**Published:** 2015-04-18

**Authors:** Norbert Gleicher, Vitaly A Kushnir, David H Barad

**Affiliations:** The Center for Human Reproduction, 21 East 69th Street, New York, NY 10021 USA; The Foundation for Reproductive Medicine, 21 East 69th Street, New York, NY 10021 USA

**Keywords:** Premature ovarian senescence (POS), Occult primary ovarian insufficiency (OPOI), Fertility counseling, Reproductive planning, Functional ovarian reserve, Fragile X mental retardation (*FMR1*) gene

## Abstract

**Background:**

Approximately 10% of women suffer from premature ovarian senescence (POS), ca. 9% as occult primary ovarian insufficiency (OPOI, also called premature ovarian aging, POA) and ca. 1% as primary ovarian insufficiency (POI, also called premature ovarian failure, POF). In a large majority of cases POS is currently only diagnosed at advanced clinical stages when women present with clinical infertility.

**Methods:**

We here, based on published evidence, suggest a new diagnostic paradigm, which is based on identifying young women at increased risk for POS at much earlier stages.

**Results:**

Risk factors for POS are known from the literature, and can be used to identify a sub-group of young women at increased risk, who then are followed sequentially with serial assessments of functional ovarian reserve (FOR) until a diagnosis of POS is either reached or refuted. At approximately 25% prevalence in general U.S. populations (and somewhat different prevalence rates in more homogenous Asian and African populations), so-called *low* (CGG_n<26_) mutations of the fragile X mental retardation 1 (*FMR1*) gene, likely, represents the most common known risk factor, including history-based risk factors from medical, genetic and family histories.

**Conclusions:**

Women so affirmatively diagnosed with POS at relative young ages, then have the opportunity to reconsider their reproductive planning and/or choose fertility preservation via oocyte or ovarian tissue cryopreservation at ages when such procedures are clinically much more effective and, therefore, also more cost-effective. Appropriate validation studies will have to precede widespread utilization of this paradigm.

## Background

The concept of fertility preservation entered medical consciousness primarily through the field of oncology, where increasingly successful chemo- and radiation therapies have improved long-term survival of young cancer patients but often result in premature ovarian senescence (POS) and indeed, frequently in outright premature ovarian failure (POF), also called primary ovarian insufficiency (POI) [1]. Healthy women, delaying childbirth for social reasons, have recently also more actively been pursuing fertility preservation (i.e., “social fertility preservation”), motivated by concerns about inadequate functional ovarian reserve (FOR) by the time they will socially be ready for conception [2,3].

A large pool of patients in need of potential fertility preservation, the approximately 10% of women who suffer from spontaneously occurring POS, have so far, however, escaped professional attention, quietly and mostly undiagnosed progressing in their POS until becoming clinically symptomatic at advanced stages of low functional ovarian reserve (LFOR) [4]. These women in a large majority of cases suffer from premature ovarian aging (POA), frequently also called occult primary ovarian insufficiency (OPOI), and in approximately only 1% of cases from POF/POI. Their presenting symptom is usually clinical infertility.

Earlier recognition of being at risk for POA/OPOI and/or POF/POI would offer this patient population the opportunity to preempt late diagnoses at already overt infertility stages and, by either changing pregnancy timing or utilizing fertility preservation techniques, would give these young women “at risk” significant opportunities at prevention of later infertility. Earlier recognition of being “at risk” can be based on historical and clinical (laboratory tests) risk factors.

Causes of POS are limited and predictable (Table 1). Only the LFOR, at times associated with endometriosis, has in the literature been proposed as a potential indication for early FOR screening [5]. Other causes of LFOR have so far failed to attract attention. Since a large majority of women affected by POS is not recognized to suffer from LFOR until presentation with infertility, these women at that point often require costly infertility treatments, which with advancing female age, in addition, decrease in effectiveness.

**Table 1:** **Known risk factors for premature ovarian senescence (POA)**

**Medical history**	**In association with**
*Conditions associated with low numbers of follicles at birth/menarche*	Turner syndrome – associated with POF/POI
	Idiopathic/genetics – association?
*Excessive recruitment*	*FMR1* mutations
	Premutation range (CGG_n=55–200_) – associated with POF/POI
	Monoalleleic *low* sub-genotype – associated with POA/OPOI
	Biallelic *low* sub-genotype – associated with POA/OPOI
	*AMHR2* gene – associated with POF/POI
	*AIRE* gene – associated with POF/POI
*Other genetic causes*	*BRCA1* mutations – associated with POA/OPOI
*Space occupying lesions and Iatrogenic factors -mostly associated with POF/POI but also with POA/OPOI*	Ovarian surgery
	Chemotherapy
	Radiation therapy
	Bone marrow transplantation
*Other medical risk factors*	Endometriosis – associated with POA/OPOI
	Polycystic ovarian syndrome (PCOS) – associated with POA/OPOI
	>>> > in association with *low FMR1* mutations and risk further augmented in presence of autoimmunity
*Autoimmunity –mostly associated with POA/OPOI but also with POF/POI*	Thyroid autoimmunity
	Adrenal autoimmunity
	Any other autoimmunity
	Autoimmune polyglandular syndromes
	Family history of autoimmune disease*
	History of repeated pregnancy loss
*Early history of maternal/sibling menopause*	

*One 1^st^ degree or two 2^nd^ degree relatives.

Age-specific follicle stimulating hormone (FSH) and/or anti-Müllerian hormone (AMH) levels have been reported, and allow objective determination of LFOR [4]. Serial longitudinal investigations of FOR in young women “at risk,” therefore, should already at relative young ages allow accurate determinations whether patients deviate from normal ovarian aging curves or not.

Absence of such prospective risk assessments in young females is the principal reason why POS is currently still only diagnosed at already advanced clinical stages. Current understanding of POS would, however, at relatively minor costs allow assessments of “risk,” and subsequent sequential longitudinal follow up with FOR test parameters like AMH, until final determination whether a patient, indeed, suffers from POS or not. Prospective FOR screening in young “high risk” patients, could, therefore, be viewed as the “PAP smear” for the detection of POS.

Such a diagnostic paradigm very rapidly would permit development of accurate, and appropriately validated, risk prediction models. Reaching similar conclusions, Cil et al. recently made the point that policy makers should integrate oocyte freezing into preventive paradigms for female infertility [6].

### Some background on ovarian aging

**Figure 1:**
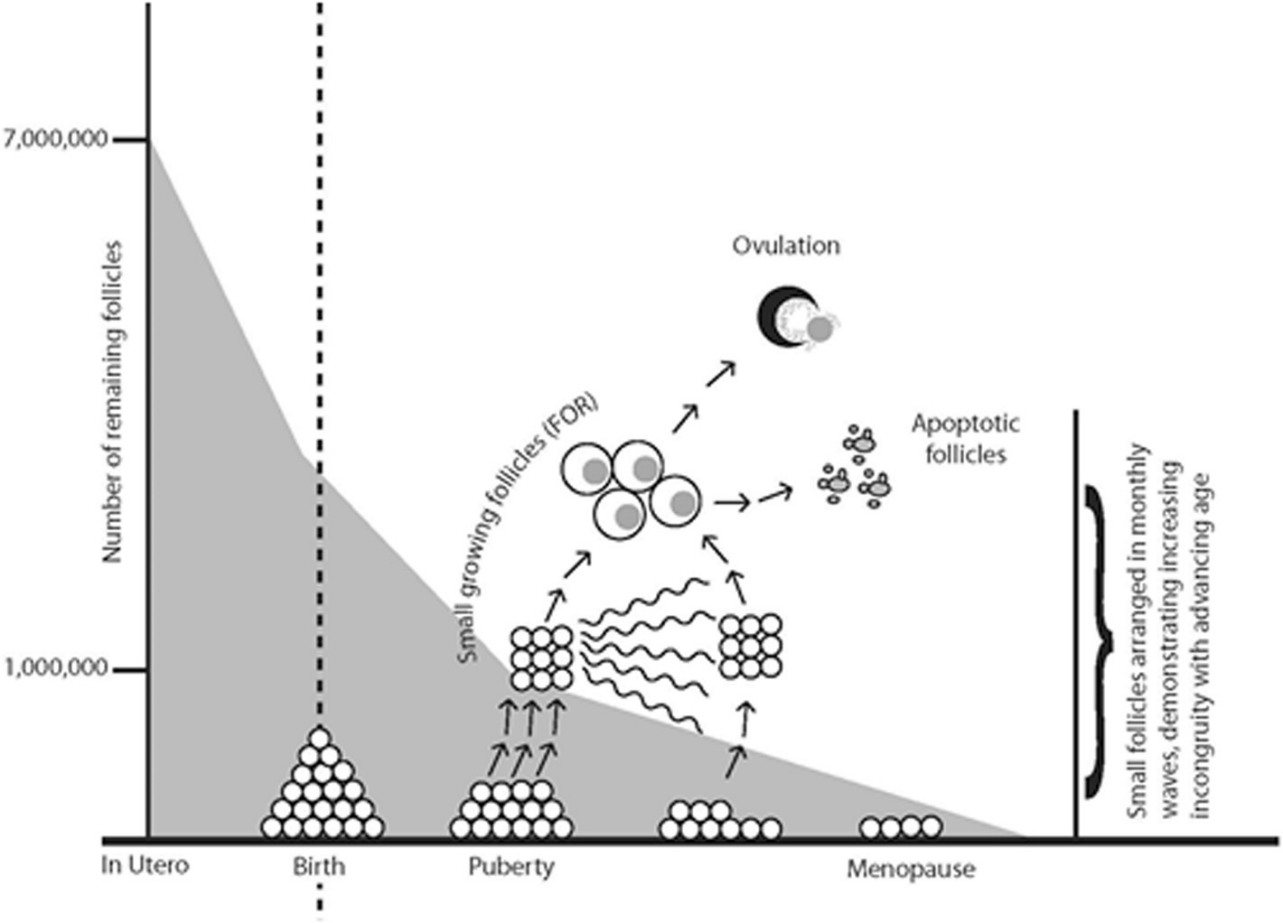
Declining follicle/oocyte numbers with advancing age.

Despite suggestions in the literature that ovarian stem cells give rise to fertilizable oocytes [7], current dogma still holds that females are born with a finite pool of follicles/eggs, which rapidly depletes during intrauterine life from a peak (~7 million follicles/oocytes), which after birth (~1 million follicles/oocytes) continues to decline at a somewhat slower pace through menarche (~400,000 follicles/oocytes) until menopause, when only a few hundred to thousands of follicles/oocytes are left in both ovaries [5]. (Figure 1).

With menarche, menstrual cyclicity is established, when the ovary transforms chaotic follicle recruitment of primordial follicles into regular, usually approximately monthly waves of developing (”growing”) follicles. A natural cycle leads to ovulation of only a single dominant follicle/oocyte. All other follicles/oocytes undergo degeneration and apoptosis during the various stages of follicle maturation, many long before the gonadotropin-sensitive stage is reached (Figure 1).

“Ovarian age” is reflected in the total ovarian reserve (TOR) of a patient. The largest part of TOR is made up of still unrecruited, “resting” primordial follicles at very primitive stages of development. “Growing” follicles are a much smaller component of TOR, and represent the so-called FOR. The FOR represents small follicles, which only weeks to months later reach maturity. Ovarian stimulation converts the unifollicular natural cycle into a polyfollicular cycle, yielding multiple follicles/oocytes [5].

Genes involved in follicle recruitment appear associated with female age at menopause. Since AMH has been suggested to play a potential role in follicle recruitment, not surprisingly, one such gene controls the AMH type II receptor (*AMHR2*) [8]. Produced by granulosa cells of small growing follicles, AMH appears to inhibit recruitment as well as subsequent follicle growth [5,9,10].

Another gene holding back recruitment is the *AIRE* gene. When mutated it leads to rapid follicle depletion and, therefore, POF/POI (see also below) [11]. It also is closely associated with control of immunologic self-tolerance. Mutations can lead to breakdown in self-tolerance and autoimmunity, placing this gene at crossroads of autoimmunity and POF/POI [12].

Genes known to affect follicle recruitment, when mutated, blocked or knocked out, primarily lead to rapid depletion of primordial follicles. Genetic control of recruitment, therefore, primarily appears to counteract a natural tendency toward explosive one-time recruitment, as still seen in more primitive water-born species.

Slow recruitment preserves follicles/oocytes at primordial stages, resulting in better TOR and FOR at later ages. This has been demonstrated in association with the *FMR1* gene, where so-called *low* alleles (CGG _n<26_) are associated with rapid depletion [13] and *high* alleles (CGG _n>34_) preserve richer FOR into advanced female ages [14].

### AMH as indicator of FOR

Speed of follicle recruitment has been reported to correlate with number of remaining primordial follicles. The growing follicle pool, representing FOR, therefore, also correlates with speed of recruitment [5,9,15]. AMH reflects this pool of small growing follicles, and is for that reason now widely considered the best laboratory tests to represent TOR [9,15]. Sample instability has, however, recently led to questions about reproducibility of results [16]. It appears that with currently commercially available AMH assays, AMH maintains clinically reasonable predictability in women with normal age-specific FOR only up to approximately age 42. In younger women with OPOI/POA AMH is of clinical value only as long as levels are not extremely low or high [17].

**Figure 2:**
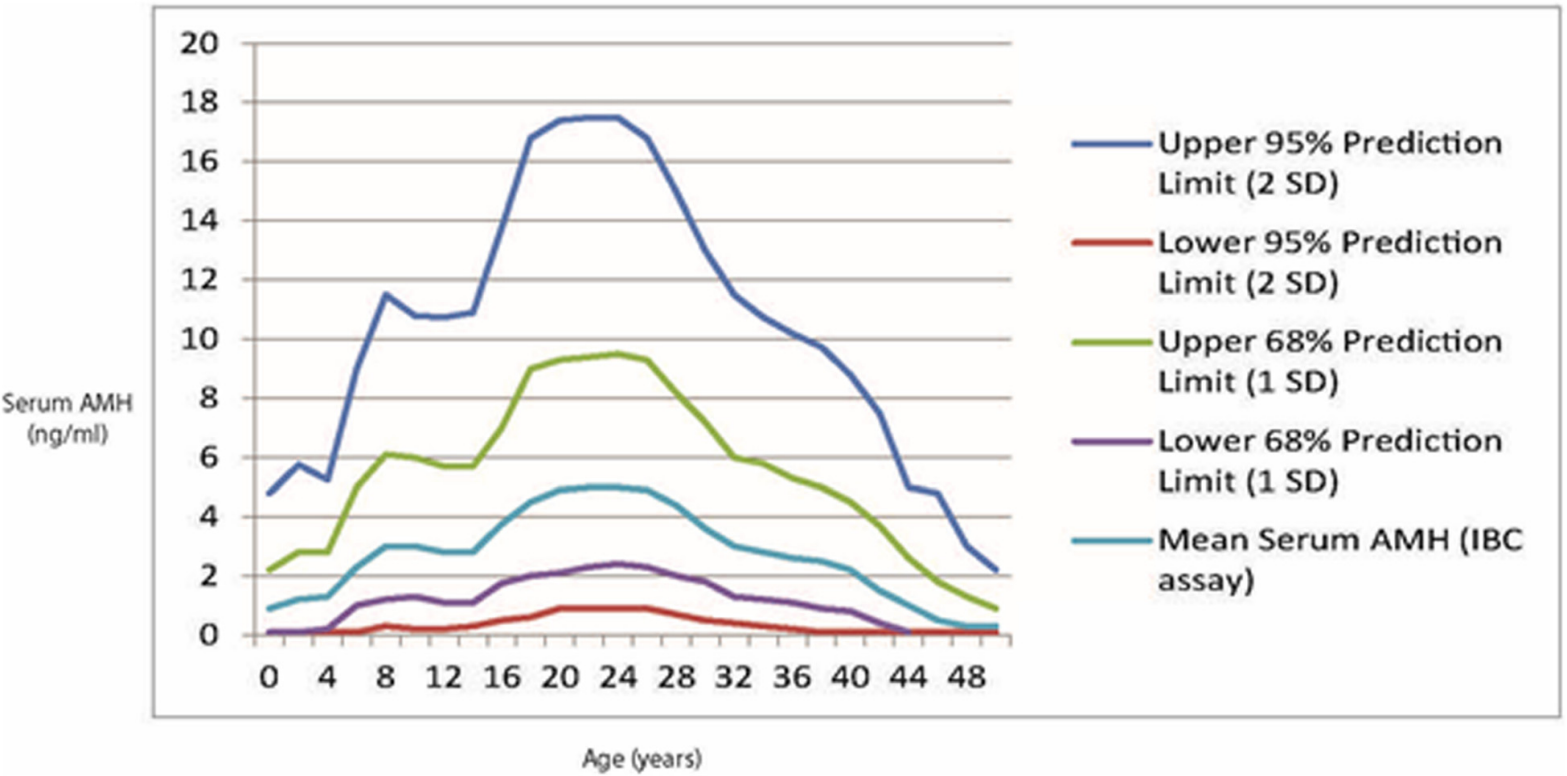
Normal age-specific AMH ranges at various prediction limits. Modified from Kelsey et al. (16), with permission.

Age-specific AMH levels have been reported by various groups, including ours [18]. Likely the most relevant study to here proposed paradigm is the one by Kelsey and associates, who, via literature searches and own data, accumulated 3,260 data points, which allowed them to define normal AMH levels in healthy premenopausal women of all ages [19]. This large database also included enough females at young ages, where fertility centers usually lack patient populations, to permit determination of prediction limits at various confidence levels (Figure 2).

The authors demonstrated that AMH levels rise from birth, reaching a peak at approximately 24–25 years. From there on, a developmental stage more familiar to fertility centers, they once again decline [19]. Available data, therefore, now permit determination of what represents normal FOR at young ages, creating the opportunity to assess whether a young female is in normal age-specific range of AMH or not.

AMH, thus, now represents a tool which allows reasonably accurate assessment of FOR and, therefore, in young women, by clinical and laboratory criteria, allows the diagnosis of likely POS (see below for definition). This is done by assessing in young women, previously determined as “at risk,” AMH longitudinally, and then establishing whether they follow a normal age-specific ovarian aging curve or not. Those who deviate from normal AMH curves into abnormally low FOR ranges can then, with considerable reliability, at still very young ages can be defined as, indeed, suffering from POS.

### Who is at risk for POS?

Since POS in all of its forms evolves insidiously as a basically asymptomatic process, OPOI/POA and POI/POF diagnoses, as noted before, are currently mostly only made at relatively late stages. Based on well-known risk factors, POS is, however, to varying degrees predictable in its occurrence (Table 1). While the statistical weight of individual predictive risk factors remain to be determined, and likely will vary in different races, as well demonstrated by the varying prevalence rates of *low FMR1* mutations among Caucasian, African and Asian women, the mere presence of any one risk factor allows for the initial designation of a young woman as “at risk”. How big the risk really is, can then be determined by the patient’s longitudinal follow up.

The PAP smear, once again, offers a good gynecologic analogy to the here proposed POS testing paradigm: As in PAP smear screening risk of cytological abnormalities and frequency of required screening is determined by patient history and PAP smear results, the ultimate risk to develop POS will be established by initial risk determination and subsequent sequential AMH testing. Those who deviate from AMH curves over relative short observation periods will be identified as, indeed, likely suffering from POS, while those following normal aging curves can be reassured.

Table 1 summarizes risk factors for POS. Risk can be the consequence of abnormally low follicle numbers at birth and/or menarche, generally believed to reflect genetic effects [4]. Turner syndrome (see also below) represents a fairly typical example, commonly characterized by premature depletion of follicles at still young ages.

POS can also be the consequence of excessive recruitment. We noted before that the *AMHR2* and *AIRE* genes are associated with excessive recruitment. Excessive recruitment has also been reported in association with *low* (CGG_n<26_) *FMR1* alleles [20]. Biallelic *low FMR1* oocyte donors already at very young ages demonstrate abnormally low FOR, while monoalleleic *low FMR1* donors at that point still demonstrate normal age-specific FOR. Over a 4-year observation period, however, even monoalleleic *low FMR1* donors already deviate in AMH levels from donors lacking *low FMR1* alleles [13]. Since *low FMR1* alleles are present in approximately 25% of all U.S. women, this population, likely, reflects the single largest risk pool for POS. An additional gene, recently associated with POA/OPOI, is the *BRCA1* gene [21].

Frequently overlooked as risk factor, are autoimmunity-associated conditions, recently reviewed [12]. Autoimmunity to thyroid often runs in parallel with autoimmunity to ovary; in principle, however, any form of autoimmunity can be associated with POA/OPOI or POF/POI. Since autoimmunity is highly familial, a family history of autoimmunity also represents risk towards POS, which may be why age at menopause is highly familial [22,23]. Early maternal and sibling ages at menopause, therefore, also should be considered risk factors towards POS. As autoimmunity can also be associated with repeat miscarriages [24], a history of repeated miscarriage should also be considered a risk factor.

Autoimmunity is believed associated at least with one-third of POS cases [25], and has been reported in humans in three distinct circumstances: Autoimmune (lymphocytic) oophoritis is only rarely observed, and practically exclusively only in presence of Addison’s disease [26]. Much more frequently, ovaries appear subject to a still poorly defined autoimmune attack, associated with thyroid autoimmunity, anti-adrenal autoimmunity and other, often non- organ-specific, autoimmune responses [26]. The increasing recognition of the X chromosome as an “autoimmune chromosome” [27] also explains the very high prevalence of associated autoimmunity with previously noted Turner syndrome [28,29].

Likely, the best-defined form of autoimmune-associated POS occurs with one of the four known autoimmune polyglandular syndromes (APS), so-called APS-1, also known as the *polyendocrinopathy candidiasis ectodermal dystrophy* or *Whitaker syndrome*. It is caused by a mutation in the previously noted autoimmune regulator (*AIRE*) gene [11]. This gene is of importance in the thymus, where it regulates self-tolerance from T cell attacks. Mutations in the gene, therefore, have been associated with attacks against “self”, i.e., autoimmunity.

A gene knockout mouse model (*AIRE* −/−) demonstrated early follicle depletion, by age 20 weeks, leading in 50-60% of animals to complete follicle depletion (POF/POI). The *AIRE* gene, therefore, is the first gene established to be associated with autoimmune-induced POS [11,30].

Other medical factors, including endometriosis and PCOS, can also suggest risk for POS. Endometriosis can cause space-occupying lesions in ovaries, frequently the target of surgeries, which reduce FOR. Endometriosis, however, is also frequently associated with autoimmunity [31]. Similarly, PCOS can be associated with autoimmunity [12], and with the previously noted *low FMR1* alleles, by themselves associated with POS [20].

Each in Table 1 listed risk factors, independently, should be considered an indication for serial FOR evaluations of young women until “risk” is either confirmed or refuted.

### A prospective screening program

What differentiates here presented screening paradigm from current practice are two crucial innovations: (i) Identification of high-risk females at very young ages (18–25 years); and (ii) Options for females confirmed as “at risk” to change their pregnancy timing and/or pursue fertility preservation.

The paradigm shift, therefore, is based on a three-step process: (i) identification of young females at increased risk towards POS via *FMR1* screening and other risk factors (ii) confirmation or refutation of increased risk by serial longitudinal monitoring with AMH (or other FOR parameters); and (iii) as early counseling and intervention as possible in cases where POS has been confirmed.

It is currently still unknown what percentage of young “high risk” females in such a screening process will be confirmed in a diagnosis of POS. Considering an approximate 10% prevalence of POS in the general population, the number of affected females should, however, be considerable. Multiple risk factors in one person can be assumed to multiply overall risk, though this, too, awaits clinical confirmation in validation studies.

**Figure 3:**
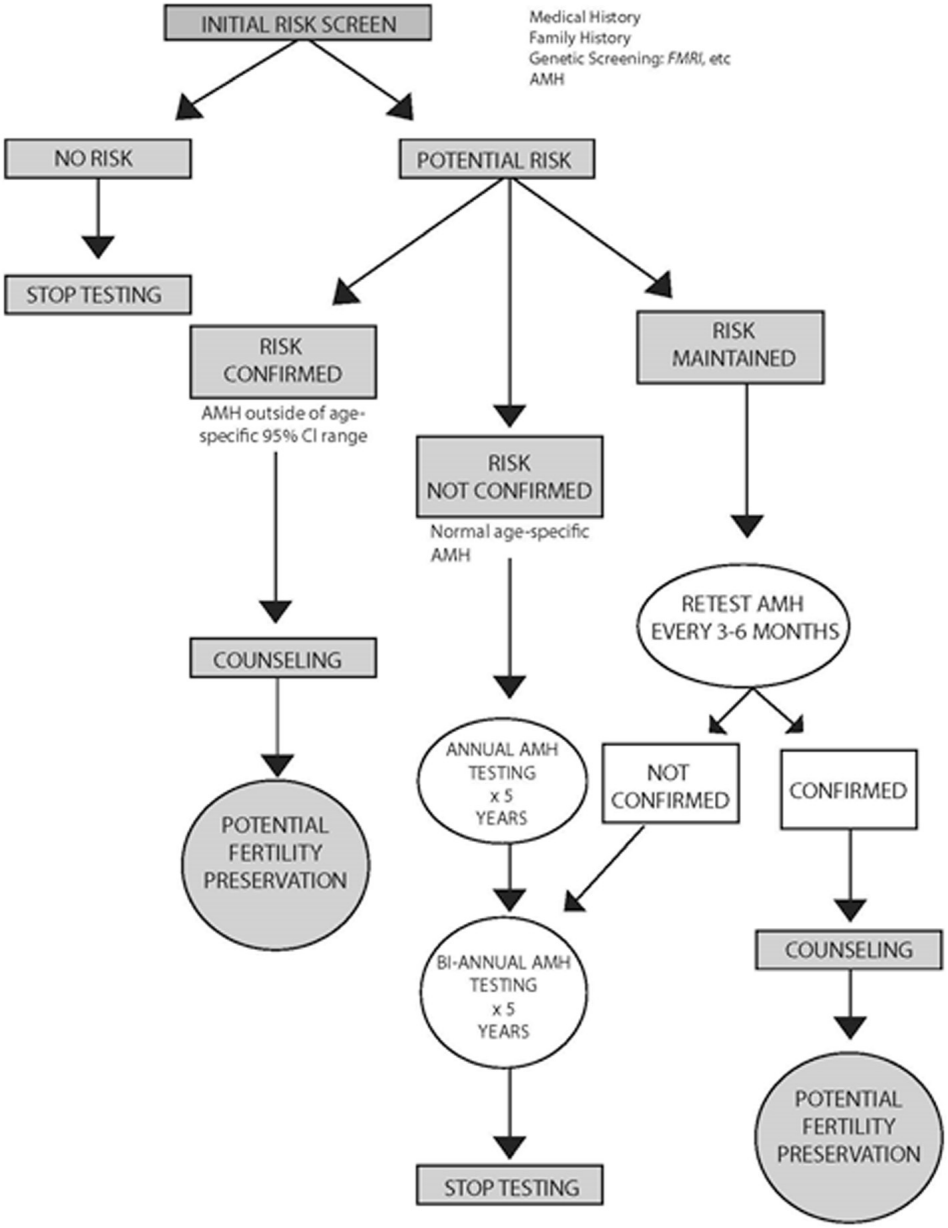
Proposed screening paradigm.

Figure 3 summarizes the proposed paradigm, and proposes a specific program for validation. While AMH results are generally considered steady [32], AMH levels can also be influenced by hormonal contraceptives [32], and do not appear as stable throughout the menstrual cycle as initially reported [33-35]. We, therefore, recommend as initial screening step two consecutive AMH evaluations, approximately one month apart.

If a patient demonstrates AMH values in age-specific mid-range, annual follow up testing should suffice. Timing can be coordinated with annual gynecological examinations, which, likely, can be switched to biannual testing after 3–5 years of no observed deviations from normal aging curves. Serial testing can, likely, be stopped the latest after approximately 10 years of follow up if no deviations from standard age-specific FOR curves have been observed. Future validation studies should quickly determine appropriate time intervals between tests and overall length of required serial testing.

Low-normal age-specific AMH values at initial evaluation or levels already outside of 95% confidence intervals for age reflect a likely POS diagnosis, requiring every 3–6 months follow up testing until, with persistently low AMH levels, a final diagnosis of POS can be reached with certainty. Abnormally high levels, often suggest polycystic ovary syndrome (PCOS) [9,15,16]. PCOS, in association with *low FMR1* alleles, also suggests increased risk of anti-ovarian autoimmunity [20] and risk towards a quickly depleting ovarian phenotype and subsequent POS [20,36]. Though POS is usually a slowly progressing process, in a minority of patients, and mostly among those with autoimmune etiology, it can proceed rapidly.

### Validation and cost effectiveness

As noted before, here proposed screening paradigm, of course, requires further validation in clinical practice. Of particular importance is the validation of individual risk factors since they, of course, will greatly vary in their respective individual impacts on risk. Because all of these validations can only be done prospectively over time, and will have to involve study populations of considerable size, they unquestionably will take time.

At the same time, one, however, also has to consider the risk of continuing to do nothing to detect POS earlier in the approximately 10 percent of women who are destined to develop POS. We would argue that the economic cost of late diagnosis with considerable certainty exceeds any potential screening costs, considering the very significant costs of fertility treatments in women POS, who in a large majority of cases, currently, are only diagnosed at rather advanced ages. In practical terms this means that the question to be asked is not whether a prospective screening process to facilitate earlier diagnosis of POS than is currently possible is cost effective but what the individual components of such a process should be to make it most cost effective.

## Conclusions

Here presented paradigm, as of this point, suggests prospective screening of selected “high-risk” females at young ages. The Eunice Kennedy Shriver National Institute of Child Health and Human Development (NICHD) and the American Society for Reproductive Medicine (ASRM) recently jointly held a conference *“to help advance the state of this science from theory to practice”* (“Ovarian Reserve: Regulations and Implications for Women’s Health, “San Diego, CA, October 2012) [37]. Here proposed new treatment paradigm offers, in the spirit of this conference, an immediately implementable change to current practice.

Finally, we in a recently accepted publication also raised the related issue of assessing risk toward POS in young women who are planning to start utilization of hormonal contraceptives for the long-term [38]. The rational here is that hormonal contraceptives suppress clinical presentations of declining fertility, like cycle irregularities; but they, in addition, affect AMH assessments [33] and, therefore prevent reliable FOR determinations. This recommendation is based on our clinical experience with a considerable number of patients who, after long periods of hormonal contraception use, suddenly found themselves diagnosed with advanced POA/OPOI or even POF/POI.

## Abbreviations

AMH: Anti-Müllerian hormone
*AMHR2*: AMH type II receptor
FOR: Functional ovarian reserve
OPOI: Occult primary ovarian insufficiency
OR: Ovarian reserve
POA: Premature ovarian aging
POF: Premature ovarian failure
POI: Primary ovarian insufficiency
POS: Premature ovarian senescence
TOR: Total ovarian reserve
